# Surgical outcomes of a prospective, phase 2 trial of robotic surgery for resectable right‐sided colon cancer (the ROBOCOLO trial)

**DOI:** 10.1002/ags3.12718

**Published:** 2023-07-19

**Authors:** Masakatsu Numata, Jun Watanabe, Atsushi Ishibe, Mayumi Ozawa, Yusuke Suwa, Keisuke Kazama, Kazuya Nakagawa, Yosuke Atsumi, Yasushi Rino, Aya Saito, Chikara Kunisaki, Itaru Endo

**Affiliations:** ^1^ Department of Surgery, Gastroenterological Center Yokohama City University Medical Center Yokohama Japan; ^2^ Department of Gastroenterological Surgery Yokohama City University Graduate School of Medicine Yokohama Japan; ^3^ Department of Surgery Yokohama City University Yokohama Japan

**Keywords:** colon cancer, complete mesocolic excision, intracorporeal anastomosis, right hemicolectomy, robotic surgery

## Abstract

**Aim:**

We evaluated the safety of robotic surgery for right‐sided colon cancer in Japan.

**Methods:**

This was a prospective, open‐label, single‐arm phase II trial conducted at two institutions. Patients ≥20 years old with stage I–III right‐sided colon cancer and scheduled for radical resection with ≥D2 lymph node dissection were eligible. The criterion for surgeons was experience performing robot‐assisted rectal resection in ≥40 cases. The primary endpoint was the postoperative complication rate ≤30 days after surgery.

**Results:**

From August 2021 to February 2023, 42 patients were enrolled; three were excluded, with 39 analyzed as the full analysis set. The median age was 72 years, and the median body mass index was 23.2. The tumor was located in the cecum in 13 cases (33.3%), ascending colon in 20 cases (51.3%), and transverse colon in six cases (15.4%). Ileocolic resection was performed in 17 cases (43.5%) and right hemicolectomy in 22 cases (56.5%), both with D3 lymph node dissection. The median console time was 109 min, and the operative time was 170 min. The mean blood loss was 7.7 mL. Intracorporeal anastomosis was performed in 28 patients (71.8%). There were no conversions and no intraoperative adverse events. The median postoperative stay was 5 days. Postoperative complications occurred in four patients (10.2%; paralytic ileus [*n* = 3] and pneumonia [*n* = 1]). All postoperative complications were grade 1 or 2, with no mortalities noted. R0 resection was achieved in all patients.

**Conclusions:**

This study demonstrated the safety and feasibility of robotic surgery for right‐sided colon cancer.

## Graphical abstract

This was a prospective, open‐label, single‐arm phase II trial, which evaluated the safety of robotic surgery for right‐sided colon cancer in Japan. This study demonstrated the safety and feasibility of robotic surgery for right‐sided colon cancer.

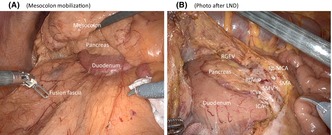

## 1 INTRODUCTION

Colorectal cancer (CRC) is the third‐most frequently diagnosed cancer and the second leading cause of cancer death worldwide.^1^ When radical surgical resection is indicated, colon resection with lymph node dissection is required.^2^ The five‐year survival rate for patients with CRC is higher than that for patients with other gastrointestinal cancers, and a long‐term survival after radical resection can be expected, so surgical techniques have a significant impact on treatment outcomes.^3^

Laparoscopic surgery, a minimally invasive surgery that can be performed with a smaller wound than open surgery and can be expected to result in early recovery after surgery, was performed in 79.9% (37 234/46 574 cases) of CRC surgeries according to a 2019 survey by the Japanese Society of Endoscopic Surgery^4^ and is also listed as a surgical treatment option in Japanese guidelines.^5^

Although laparoscopic surgery for CRC has advantages of being minimally invasive, with minimal blood loss and rapid postoperative recovery, laparoscopic right colectomy (Lap‐RC) and optimal lymph node dissection can cause complications, such as venous injury to the superior mesenteric vein and accessory right colic vein and pancreatic injury, which can lead to a serious condition in the patient. According to the National Clinical Database in Japan, the surgical mortality rate of right hemicolectomy (RHC) has been higher than that of low anterior resection at approximately 2% over the past 10 years and is not improving.^6^ These data indicate that RHC needs to be developed into a safer and more reliable procedure.

Robotic systems have introduced advanced technologies, such as integrated three‐dimensional (3D) visualization and improved dexterity, which allow for more accurate dissection and are expected to improve the safety of right colectomy (RC). Since the first report of robotic RC (Ro‐RC) in 2002, international literature has shown that Ro‐RC can be performed with comparable short‐ and long‐term outcomes to conventional laparoscopic surgery.^7^, ^8^, ^9^, ^10^ In Japan, robotic surgery for rectal cancer was first covered by national health insurance in 2018, and colon surgery was covered by insurance in 2022. Currently, Ro‐RC is gradually expanding in Japan, but only one prospective clinical trial has been reported to date.^11^

In this prospective phase II study, we evaluated the safety of Ro‐RC in the introduction phase.

## 2 PATIENTS AND METHODS

### 2.1 Trial design and participants

**FIGURE 1:**
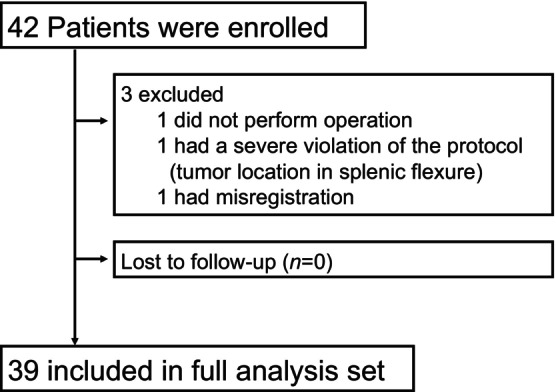
Consort diagram of patient registration.

This study was a prospective, multicenter, open‐label, single‐arm phase II trial at two institutions (Figure 1). The study protocol was approved by the clinical research review board of Yokohama City University (CRB3180007) and the institutional review board of each participating hospital before the study was initiated. All patients provided their written informed consent before enrolling in the study. This study was registered with the Japan Registry of Clinical Trials (jRCT1032210196).

Eligibility criteria were as follows: (1) histologically proven primary adenocarcinoma located at either the cecum, ascending colon, or right transverse colon; (2) scheduled to undergo either ileocecal resection (ICR), partial colectomy (cecum, ascending colon) (PC (C, A)), or RHC, with either D2 or D3 lymph node dissection; (3) clinically diagnosed with Union for International Cancer Control (UICC) TNM classification (8th edition)^12^ clinical stage I‐IIIC (T1–T4b, N0–N2b, M0); (4) tumor diameter ≤8 cm; (4) ≥20 years old; and (5) Eastern Cooperative Oncology Group Performance status (ECOG‐PS) of 0 or 1. The exclusion criteria were as follows: (1) a history of chemo‐ or radiotherapy for a previous cancer; (2) a history of laparotomy (except for appendectomy and caesarian section); (3) a history of laparoscopic gastrectomy or colorectal resection; (4) presence of either hematological, liver, or renal dysfunction; and (5) synchronous multiple cancer requiring multiple anastomoses. Complete inclusion and exclusion criteria are provided in Supplement 1.

### 2.2 Treatment

In this trial, the surgeon's criteria were set according to the protocol (a total of three colorectal surgeons participated who had a qualified surgeon with endoscopic surgical skills endorsed by the Japan Society of Endoscopic Surgery and had performed ≥40 cases of robotic‐assisted rectal cancer surgery).

**FIGURE 2:**
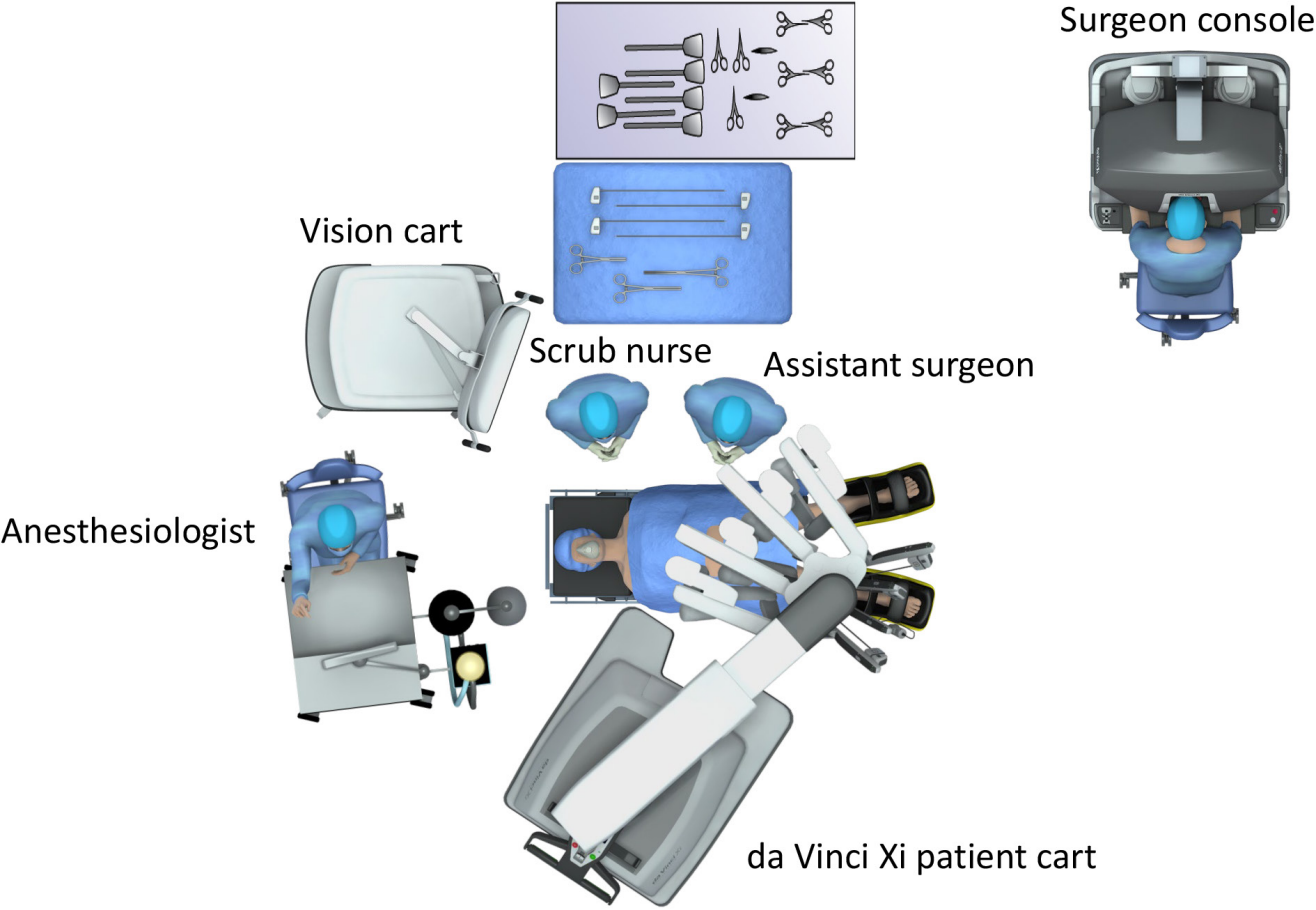
The operation room setup.

**FIGURE 3:**
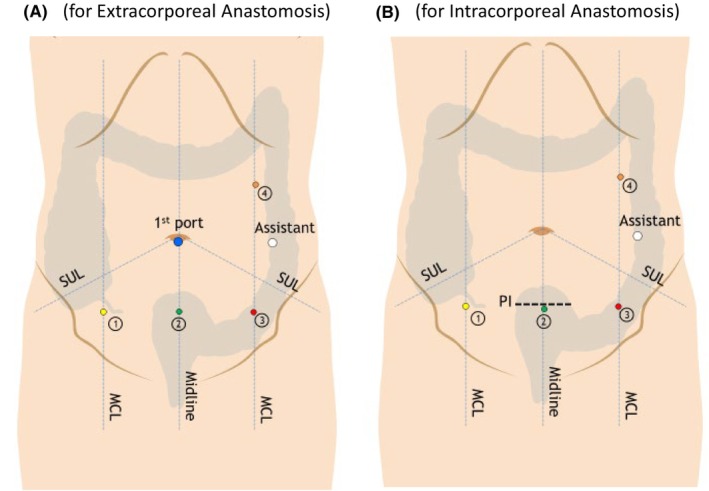
Port placement (A, extracorporeal anastomosis; B, intracorporeal anastomosis). 1, fenestrated bipolar forceps; 2, endoscope; 3, monopolar scissors or vessel sealers; 4, tip‐up fenestrated graspers. MCL, midclavicleline; PI, Pfannenstiel incision; SUL, supine‐umbilical line.

The operating room setup is shown in Figure 2. In all cases, Ro‐RC was performed using the da Vinci Surgical System Xi (Intuitive Surgical). Typical port arrangements are shown in Figure 3 (A,B: extracorporeal and intracorporeal anastomosis). The instruments typically used were monopolar scissors, fenestrated bipolar forceps, tip‐up fenestrated graspers, and vessel sealers.

**FIGURE 4:**
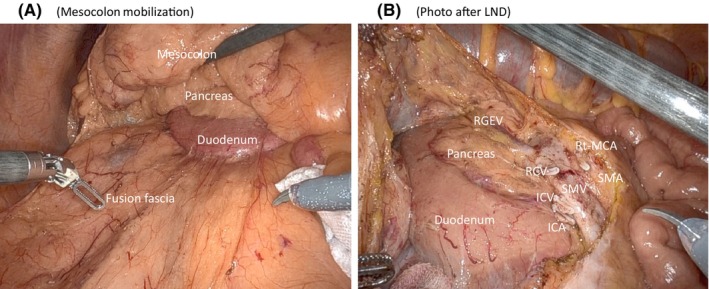
The operative photograph after mesocolon mobilization (A) and after D3 lymph node dissection (B). ICA, ileocolic artery; ICV, ileocolic vein; RCV, right colic vein; RGEV, right gastroepiploic vein; Rt‐MCA, right branch of middle colic artery; SMA, superior mesenteric artery; SMV, superior mesenteric vein.

Regarding the general procedure for Ro‐RC, colonic mobilization was first performed by a caudal retroperitoneal approach (Figure 4A). Lymph node dissection with vascular ligation was then performed (D2 or D3 dissection). The vessels ligated were the ileocolic artery, right colic artery, and middle colonic artery, depending on the location of the tumor (Figure 4B). With regard to the surgical procedure, we defined ICR as dissection of the ileocolic artery only, PC (C, A) as dissection of the ileocolic artery and the right colonic artery, and RHC as dissection of the ileocolic artery, right colonic artery and middle colonic artery (including dissection of the right branch only). D2 dissection was performed up to the right margin of the superior mesenteric vein (SMV), and D3 dissection was performed up to the left margin of the SMV. In cases of transverse colon cancer, No. 223 dissection was performed up to the left lateral margin of the superior mesenteric artery (SMA), including the area around the root of the middle colic artery (MCA). For bowel resection, the proximal and distal sides of the bowel were resected with a margin of 10 cm from the tumor, and blood flow was confirmed by the indocyanine green (ICG) fluorescence method. Reconstruction was performed by intracorporeal anastomosis (IA) or extracorporeal anastomosis (EA), at the surgeon's discretion. The method of IA was not specified in the protocol. For EA, the specimen was removed through a small incision at the umbilicus, and anastomosis was performed, and for IA, the specimen was removed via the Pfannenstiel incision.

### 2.3 Perioperative care

Patients were scheduled to be hospitalized the day before surgery. Mechanical and chemical bowel preparation was performed preoperatively. Mechanical pretreatment consisted of sodium picosulfate hydrate (0.75%/10 mL) and high‐concentration magnesium citrate solution (34 g/180 mL) taken internally at 2 p.m. the day before surgery. Chemical pretreatment consisted of metronidazole 750 mg and kanamycin 1000 mg taken orally at 5 p.m. and 9 p.m. on the day before surgery. One prophylactic antibiotic (Flomoxef; Shionogi, Osaka, Japan) was administered at the induction of anesthesia, with an additional dose 3 h after surgery if necessary. The nasogastric tube was removed immediately after surgery. Postoperative oral liquid intake is usually permitted the day after surgery, and a normal diet is resumed on the second or third postoperative day in cases with an uneventful postoperative course, but these are not specified in the protocol and are left to the discretion of each institution. The intravenous catheter was removed when enteral feeding was possible without nausea or vomiting. Mobilization of patients was started from the first postoperative day. The criteria for discharge were as follows: (1) recovery of the bowel function, as evidenced by abdominal radiography and flatus/defecation status; (2) adequate food intake achieved; (3) no or well‐controlled pain; (4) postoperative complications, if any, improved or brought under control, with the patient understanding their condition and consenting to discharge.

### 2.4 Endpoints and statistical analyses

The primary endpoint was the incidence of postoperative complications within 30 days after surgery. Postoperative complications are defined as all adverse events that occur and are judged to be “causally related” to the study. In this study, all postoperative complications of any grade occurring within 30 days after surgery, including those occurring after discharge from the hospital, were included. The following secondary endpoints were also evaluated: (1) conversion, (2) readmission within 30 days after surgery, (3) mortality within 30 days after surgery, (4) intraoperative adverse events (vascular injury/organ damage/retry of bowel reconstruction/other), (5) postoperative recovery (hospital stay, days to first flatus, days to oral intake), and (6) surgical outcomes (intraoperative blood loss, overall operation time, console time, lymph nodes harvested, R0 resection rate).

According to the Japanese National Clinical Database (NCD) in 2014–2018,^13^ the RELARC study reported in 2021,^14^ the COLD study,^15^ and Bouno et al.,^16^ the postoperative complication rate within 30 days after Lap‐RC ranged from 18% to 47%. Previous reports of Ro‐RC for resectable right‐sided colon cancer have reported postoperative complication rates of 7.6%–21.6% within 30 days after surgery.^7^, ^17^, ^18^, ^19^ Therefore, we estimated that the expected rate of postoperative complications within 30 days after Ro‐RC is approximately 16%. If the postoperative complication rate of Ro‐RC does not exceed the threshold rate of 30% for Lap‐ RC, the safety of Ro‐RC can be demonstrated. Assuming an expected postoperative complication rate of 16% (84% complication‐free rate) within 30 days after Ro‐RC, a threshold of 30% (70% complication‐free rate), a one‐sided α of 5%, and 70% power, the sample size was set at 41 patients to account for 5% of required cases being excluded or dropping out when calculated using the optimal method of Simon's two‐stage phase II design.^20^

The primary analysis population was a full analysis set (FAS) for which at least one dose of protocol therapy was administered, all selection criteria were met, no exclusion criteria were violated, and some data were available. If nine or more cases of postoperative complications occurred within 30 days of surgery overall, the study treatment was to be rejected.

All statistical analyses were carried out using the SAS software program, version 9.4 (SAS Institute Inc., Cary, NC, USA), and the EZR and GraphPad Prism software programs, version 6.01, for Windows (GraphPad Software, San Diego, CA, USA).

## 3 RESULTS

### 3.1 Patients' characteristics

From August 2021 to February 2023, 42 eligible patients were registered from two institutions. Three patients were excluded because of operation postponement (*n* = 1), violation of eligibility criteria (tumor location in splenic flexure; *n* = 1), and misregistration (*n* = 1); consequently, 39 patients were therefore subjected to the FAS analysis (Figure 4).

The details of the FAS population are presented in Table 1. The median age and body mass index were 72 years old and 23.2, respectively. All patients had an ECOG‐PS 0, and 28 (71.7%) patients had some comorbidity. Thirteen patients (33.3%) had tumors located in the cecum, 20 (51.3%) in the ascending colon, and six (15.4%) in the transverse colon; 21 (53.8%) were diagnosed as clinical stage 1, eight (20.5%) as clinical stage 2, and 10 (25.6%) as clinical stage 3.

**TABLE 1:** Patient characteristics of full analysis set.

Parameters	Category	(*n* = 39)
Age (years)		72 (42–85)
Sex	Male	18 (46.2%)
	Female	21 (53.8%)
BMI (kg/m^2^)		23.2 (15.8–34.9)
ECOG‐PS	0	39 (100.0%)
ASA‐PS	1	3 (7.7%)
	2	34 (87.2%)
	3	2 (5.1%)
Comorbidity	Any	28 (71.7%)
	Cardiovascular	17 (43.6%)
	Respiratory	3 (7.7%)
	Diabetes	7 (17.9%)
	Renal	4 (10.3%)
	Metabolic	8 (20.5%)
Serum Albumin		4.2 (2.9–4.9)
Abdominal surgery history		9 (23.1%)
Tumor location	Cecum	13 (33.3%)
	Ascending	20 (51.3%)
	Transverse	6 (15.4%)
cStage	1	21 (53.8%)
	2	8 (20.5%)
	3	10 (25.6%)

Abbreviations: ASA‐PS, American society of anesthesiology performance status; BMI, body mass index; c, clinical; ECOG‐PS, Eastern cooperative oncology group performance status.

### 3.2 Surgical outcomes

The operative outcomes are shown in Table 2. ICR was performed in 13 patients (33.3%), PC (C, A) in four patients (10.3%), and RHC in 22 patients (56.5%), accompanied by D3 lymph node dissection in all patients. The median total console time and total operation time were 109 and 170 min, respectively. The mean blood loss was 7.8 mL, with a maximum of 61 g. IA was performed in 28 patients (71.8%), including IA‐Delta anastomosis in 15 (38.5%), IA‐Overlap in 10 (25.6%), and IA‐functional end‐to‐end anastomosis in three (7.7%). No conversion and no intraoperative adverse events, such as vascular injury, organ damage, or retry of bowel reconstruction, were recorded. The median postoperative stay was 5 days.

**TABLE 2:** Perioperative findings.

Parameters	Category	(*n* = 39)
Procedure	ICR	13 (33.3%)
	PC (C, A)	4 (10.3%)
	RHC	22 (56.4%)
LND	D3	39 (100.0%)
Console time (min)		109 (40–272)
Operation time (min)		170 (102–356)
Blood loss (g)		0 (0–61)
Anastomosis	IA	28 (71.8%)
	IA‐Delta	15 (38.5%)
	IA‐Overlap	10 (25.6%)
	IA‐FEEA	3 (7.7%)
	EA	11 (28.2%)
	EA‐FEEA	11 (28.2%)
Conversion		0 (0.0%)
Intraoperative AE		0 (0.0%)
Days to flatus		1 (1–9)
Days to Diet		2 (2–9)
Postoperative hospital Stay		5 (5–15)

Abbreviations: AE, adverse event; EA, extracorporeal anastomosis; FEEA, functional end to end anastomosis; IA, intracorporeal anastomosis; ICR, Ileocecal resection; LND, lymph node dissection; PC (C, A), Partial colectomy (cecum, ascending colon); RHC, right hemicolectomy.

The postoperative complication details are summarized in Table 3. Postoperative complications occurred in four cases (10.2%), which did not exceed the nine cases specified in the study protocol, so the primary endpoint was met. Of the four cases, one had a Clavien–Dindo^21^ Grade of 1, and three had a Grade of 2. For grade 1 paralytic ileus (*n* = 1, 2.5%), the diet was postponed a few days due to abdominal retention complaints, which quickly recovered without any medication. Regarding the grade 2 complications, the postoperative paralytic ileus (*n* = 2, 5.1%) and pneumonia (*n* = 1, 2.5%) fully recovered after nasal tube insertion and antibiotic medication, respectively.

**TABLE 3:** Morbidity detail.

CD grade	Category	(*n* = 39)
All‐grade	Total	4 (10.2%)
Grade 1	Total	1 (2.5%)
	Paralytic ileus	1 (2.5%)
Grade 2	Total	3 (7.6%)
	Paralytic ileus	2 (5.1%)
	Pneumonia	1 (2.5%)
Grade 3		0 (0.0%)
Grade 4		0 (0.0%)
Grade 5		0 (0.0%)

Abbreviation: CD, Clavien–Dindo.

Table 4 shows the pathological findings. Overall, 13 (33.3%), three (7.7%), and two (5.1%) of the patients had pathological T3, T4a, and T4b, respectively. Sufficient proximal (128 mm) and distal margins (110 mm) and R0 resection were achieved in all patients. The median number of harvested lymph nodes was 22.

**TABLE 4:** Pathological findings.

Parameters	Category	(*n* = 39)
Histological type	Tub	37 (94.8%)
	Por	2 (5.2%)
pT	T1	15 (38.5%)
	T2	6 (15.4%)
	T3	13 (33.3%)
	T4a	3 (7.7%)
	T4b	2 (5.1%)
pN	N0	32 (82.1%)
	N1	4 (10.3%)
	N2	3 (7.7%)
Proximal margin (mm)		128 (42–365)
Distal margin (mm)		110 (25–500)
R0 resection		39 (100.0%)
Harvested LN		22 (3–56)

Abbreviations: LN, lymph node; p, pathological; por, poorly differentiated adenocarcinoma; Tub, tubular adenocarcinoma.

## 4 DISCUSSION

The study in the present cohort demonstrated the safety and feasibility of Ro‐RC for right‐sided colon cancer in the introduction phase in Japan.

The laparoscopic approach for patients with CRC has expanded worldwide over the past two decades. However, technical difficulties associated with performing this procedure, particularly with respect to optimal lymph node dissection, have been reported for right‐sided CRC.^22^, ^23^, ^24^ One possible reason for this is that the procedure can cause bleeding due to damage to the tributaries of the superior mesenteric vein, which has complex vascular variations, and pancreatic injury.^25^

Although several laparoscopic approaches have been demonstrated,^26^, ^27^ no gold standard has yet been established for the entire process of lymph node dissection. In fact, according to the 2020 Japanese National Clinical Database Annual Report, the rate of laparoscopic surgery for RHC is only 54.2%, which is low compared to other CRC procedures. The report also found that the postoperative 90‐day mortality rate for RHC is 2.2%, which is approximately 4 times higher than that for low anterior rectal resection.^6^

The advantages of robotic surgery over conventional laparoscopic surgery include a stable 3D view and improved dexterity using multi‐wrist instruments, which may help ensure secure lymph node dissection along the SMV and dissection between the mesocolon and the duodenum/pancreas. Robotic platforms are expected to reduce intraoperative adverse events and provide an additional level of safety.

However, while several studies in Europe and the United States have reported on the short‐term outcomes of Ro‐RC, there is little solid evidence to date to support Ro‐RC because of the small number of cases and the retrospective design of the studies performed. Previous reports have found morbidity rates ranging from 10% to 29% and mortality rates ranging from 0% to 3% after Ro‐RC. In Korea, Park et al. randomized 70 patients (35 in each group) for Ro‐RC and Lap‐ RC and reported an overall grade morbidity of 17.1% and 20.0% for Ro‐RC and Lap‐RC, respectively, with no mortality in either group. Complications in the Ro‐RC group were anastomotic leak (AL) in one case (2.8%) and intraluminal bleeding requiring transfusion in one case (2.8%).^10^ Dohhn et al. performed propensity matching comparing 359 Ro‐RC and 718 Lap‐RC cases using the Danish national database from 2014 to 2018. The total rate of postoperative complications in the Ro‐RC group was 21.7%, with AL experienced by 3.1%, and the 30‐day mortality was 2.2%.^9^ In addition, a systematic review including 15 studies comparing Ro‐RC and Lap‐RC found that the morbidity and mortality rates for Ro‐RC were 10%–29% and 0%–3.3%, respectively, and were not significantly different from those of Lap‐RC.^28^ There has been only one report in Japan to date, showing Clavien–Dindo grade II or higher morbidity of 11%, grade III morbidity of 4% and no mortality after Ro‐RC for right‐sided colon cancer.^11^ In this study, the morbidity of Clavien–Dindo grade II or higher was 7.6%, grade III was 0%, and there was no mortality, similar to previously reported results.

The present study showed relatively favorable results, and the all‐grade morbidity and mortality rates were 10.2% and 0%, respectively. This can be attributed, at least in part, to the fact that we experienced no cases of AL. According to a systematic review of 15 previous articles, the rate of AL following Ro‐RC ranges from 0% to 4%.^28^ Another study reported by Morpurgo et al. compared Ro‐RC with IA against Lap‐RC with EA, noting a significantly lower AL rate following Ro‐RC (0% vs. 8.3%).^29^ Generally, robotic platforms are often accompanied by ICG fluorescence bowel perfusion confirmation,^30^ and the reduced bowel traction during the IA procedure^31^ may contribute to the low AL rate in Ro‐RC.

The present study also highlights the positive aspect of robotic surgery, including the low rate of intraoperative adverse events. One fatal intraoperative adverse event associated with RC is major bleeding from the fragile tributaries of the SMV due to excessive intraoperative traction, as excessive blood loss during surgery contributes to postoperative morbidity and mortality.^32^ Previous studies regarding intraoperative adverse events were limited due to their retrospective settings. Prospective data from 132 cases assessed by Di Buono et al. showed three (2.2%) intraoperative adverse events during Lap‐RC, all of which were bowel perforation.^15^ The present study categorized intraoperative adverse events into four categories of vascular injury, organ injury, retry of bowel reconstruction, and others. Our data revealed no intraoperative adverse events, including vascular injury requiring intraoperative blood transfusion, and the maximum blood loss was 62 mL. Organ damage was also not recorded. Based on these findings, the conduct of a stabilized procedure thanks to the use of robotic technologies may contribute to a reduction in intraoperative handling failure.

In the present study, the operating surgeons were limited to those with operating experience in performing robot‐assisted rectal cancer resection for ≥40 cases. A previous study showed that the number of cases required to perform in order to master the learning curve was approximately 20,^33^ suggesting that the three surgeons participating in our study had a relatively good level of general operating skill in robotic surgery, which may also have contributed to the reduction in the rates of intraoperative adverse events and conversion. Although robotic surgery has been reported to require a longer operative time than laparoscopic surgery, the median total operative time was 170 min, which is in the range of previous cases of Lap‐RC.^8^, ^34^

Several limitations associated with the present study warrant mention. First, since only those with sufficient robotic surgery experience were selected as surgeons in this study, the results of this study may not be generalizable, especially for surgeons in the early stages of the learning curve. Second, the present study included Japanese patients, who tend to have a lower body mass index than patients in Western countries. The present results therefore cannot be directly compared to those of Western studies. Third, although the required sample size was calculated using a statistical model, this study was not based on a large cohort. Fourth, since this study was not a randomized trial, our cohort may have selection bias. Fifth, robotic ICR was performed in one‐third of the cases in the present study. Therefore, surgical outcomes may tend to be better than if robotic RHC had been performed in all cases. Sixth, the oncological long‐term outcome was not evaluated in the present study.

In summary, this study demonstrated the safety and feasibility of Ro‐RC for right‐sided colon cancer during the introduction period in Japan. Currently, Ro‐RC is predominantly performed in limited institutes in Japan. With increases in availability, consensus discussion now exists regarding whether robotic platforms would translate to patient and economic benefits. Future studies are needed to establish Ro‐RC as an optimal procedure for maximal surgical safety.

## AUTHOR CONTRIBUTIONS

Authors making substantial contributions to conception and design: JW, acquisition of data: MN, JW, AI, MO, YS, KK, KN, YA, YR, AS, CK, and IE. Authors participating in drafting the article: MN and JW. Authors participating in drafting the article or revising it critically for important intellectual content: MN, JW, AI, MO, YS, KK, KN, YA, YR, AS, CK, and IE. All authors read and approved the final manuscript.

## FUNDING INFORMATION

None.

## CONFLICT OF INTEREST STATEMENT

The authors declare no conflicts of interest for this article. The authors, Jun Watanabe and Itaru Endo, are editorial members of the *Annals of Gastroenterological Surgery*.

### DATA AVAILABLITY STATEMENT

The collected data or related documents will not be made available to others.

## ETHICS STATEMENT

The ROBOCOLO trials were conducted in accordance with the Declaration of Helsinki, the Japanese Ethical Guidelines for Medical and Health Research Involving Human Subjects, and the Clinical Trial Acts in Japan. Each trial was approved by the Yokohama City University Certified Review Board.

Approval of the research protocol: N/A.

Informed consent: Written informed consent was obtained from all patients before enrollment.

Registry and the Registration No. of the study/trial: N/A.

Animal Studies: N/A.

## Supporting information


Supplement 1.
