# Supplementary material for: Surgical outcomes of a prospective, phase 2 trial of robotic surgery for resectable right‐sided colon cancer (the ROBOCOLO trial)
Source: Ann Gastroenterol Surg. 2023 Jul 19;8(1):80–7. doi: 10.1002/ags3.12718 (PMC10797943; doi:10.1002/ags3.12718)
Supplement: Supplementary file 1 — Supplement 1. [file AGS3-8-80-s001.docx]

**Supplement 1: Inclusion/ Exclusion Criteria**

**Inclusion criteria:**

**(Patient Selection Criteria)**

(1) Patient age ≥ 20 years old at the time of enrollment.

(2) Patients with a histological diagnosis of colon cancer based on endoscopic biopsy.

(3) Patients who have been diagnosed as being amenable to radical resection based using various preoperative diagnostic imaging.

(4) Patients whose main site of tumor is either the cecum (C), ascending colon (A), or right-sided transverse colon (T), and who are scheduled to undergo right hemicolectomy of the colon with lymph node dissection of D2 or greater

(5) Clinical stage I to IIIC (T1 to 4b, N0 to 2b, and M0, according to TNM classification)

(6) Patients diagnosed as not indicated for endoscopic resection.

(7) Tumor diameter ≤ 8 cm.

(8) No multiple lesions requiring more than two anastomotic sites.

(9) Eastern Cooperative Oncology Group Performance status score between 0 and 1.

(10) Patients who have sufficient judgment to fully understand the study content.

(11) Patients who have obtained written informed consent for participation in the study.

**(Surgeon Selection Criteria)**

(1) Surgeons who have performed at least 40 cases of robot rectal resection.

**Exclusion criteria**

(1) A history of chemotherapy (systemic anticancer drug therapy, radiation therapy) including treatment for other types of cancer

(2) A history of laparotomy (excluding appendectomy and Cesarean section)

(3) A history of laparoscopic gastrectomy or colorectal resection

(4) Patients with the following latest laboratory values within 14 days prior to enrollment

White blood cell count <3000/mm3

Platelets <75,000/mm3

Hemoglobin <7 g/dL

AST > 100 IU/L

ALT > 100 IU/L

Serum Cr>1.5 mg/dL

(5) Other patients deemed unsuitable by the investigator
